# Intensive plaque modification with rotational atherectomy and cutting balloon before drug-eluting stent implantation for patients with severely calcified coronary lesions: a pilot clinical study

**DOI:** 10.1186/s12872-016-0273-8

**Published:** 2016-05-26

**Authors:** Qiyong Li, Yong He, Li Chen, Mao Chen

**Affiliations:** Department of Cardiology, West China Hospital, Sichuan University, Chengdu, China; Department of Cardiology, Sichuan Provincial People’s Hospital & Sichuan Academy of Medical Science, Chengdu, China; Department of Physiology, West China School of Preclinical and Forensic Medicine, Sichuan University, Chengdu, China

**Keywords:** Coronary calcification, Rotational atherectomy, Cutting balloon, Randomized controlled trial

## Abstract

**Background:**

This study investigated whether, for patients with severely calcified coronary lesions, use of a cutting balloon (CB) during rotational atherectomy (RA) before placing a drug-eluting stent will improve periprocedural outcomes, compared to RA with a conventional plain balloon.

**Methods:**

In a randomized controlled trial, patients with severely calcified lesions of calcium arc ≥180° were apportioned to receive intensive plaque modification with RA and CB (RA + CB; *n* = 35) or RA with conventional plain balloon (RA; *n* = 36). Intravascular ultrasound was applied for quantitative or qualitative analyses of percutaneous coronary intervention outcomes. The primary outcome was acute lumen gain after drug-eluting stent.

**Results:**

The RA + CB and RA groups were similar in baseline mean arcs of superficial calcium, and minimum lumen cross-sectional areas (CSAs). The mean minimum stent CSA after percutaneous coronary intervention (PCI) of the RA + CB group (5.9 ± 1.7 mm^2^) was significantly larger than that of the RA group (5.0 ± 1.4 mm^2^; *P* = 0.021). Patients in the RA + CB group achieved significantly larger acute CSA gain after PCI (4.5 ± 1.5 mm^2^) relative to the RA group (3.8 ± 1.5 mm^2^; *P* = 0.035). The groups were similar in rates of periprocedural complications, but at the 1-year follow-up the RA + CB had a lower rate of revascularization for restenosis of the target vessel and MACE (5.7 %) than did the RA group (22.2 %, *P* = 0.046).

**Conclusion:**

Aggressive plaque preparation with RA and CB seems to be safe and effective for patients with severely calcified coronary lesions.

**Trial registration:**

Current Controlled Trials ChiCTR-INR-16008274. Retrospectively registered 12 April 2016.

## Background

Calcification of the coronary lesion is a clinically important characteristic of coronary atherosclerosis that responds poorly to conventional percutaneous coronary intervention (PCI) [1, 2]. Indeed, severely calcified coronary lesions are challenging for interventional cardiologists during the PCI process since they can cause the balloon dilation to fail and subsequent incomplete and asymmetrical stent expansion [3, 4]. Calcified coronary lesions are also associated with increased risk of adverse events after PCI, such as stent restenosis and thrombosis [5–8]. Thus, coronary calcification warrants the development of further treatment strategies for patients with these lesions.

During the past few decades, many novel devices and techniques have been proposed to treat severely calcified coronary lesions [9, 10]. The results of early observational studies and clinical trials suggested that modification of severely calcified coronary lesions with rotational atherectomy (RA) may ease the process of angioplasty and PCI [11, 12]. Other clinical trials indicated that pre-modification of the calcified coronary lesions with RA improved acute periprocedural outcomes, such as acute gain of the lumen [13, 14], with relatively few major adverse cardiovascular events (MACE) [15]. In view of this, the guidelines for PCI of the 2011 American Heart Association, and the American College of Cardiology, recommend RA as an optional therapy for patients with severely calcified coronary lesions (Class IIa) [16]. Moreover, to further improve the clinical application of RA, a standardized protocol has recently been published by a European expert consensus [17]. However, a clinical trial published in 2013 called into question the benefit of routine lesion preparation using RA, as it was not associated with an improved mid-term clinical outcome [18]. RA may not be adequate for some patients with severe coronary calcification.

The cutting balloon (CB) has been proposed as a treatment for calcified coronary lesions, but clinical evidence is limited to a few case reports [19, 20] or observational studies [21]. The application of the CB for angioplasty has proved successful for treating hemodialysis access stenosis, and the 6-month patency achieved with the CB was significantly higher compared with balloon angioplasty [22]. These facts suggest that in cases of severely calcified coronary lesions, plaque modification with both RA and CB before drug-eluting stent implantation may improve the PCI. Although a retrospective study implied that RA combined with CB could have a favorable role in lesion preparation [23], to the best of our knowledge, there have been no relevant randomized controlled trials published.

Therefore, we conducted a pilot randomized controlled trial to evaluate the efficacy and safety of intensive plaque modification with RA and CB angioplasty before placement of a drug-eluting stent, relative to that of RA with a plain conventional balloon. The periprocedural outcomes of patients with severely calcified coronary lesions were confirmed on intravascular ultrasound (IVUS).

## Methods

### Patients and study protocol

This study was designed as a two-center open-label randomized controlled study to evaluate the safety and efficacy of RA combined with cutting balloon (CB) angioplasty, relative to conventional balloon angioplasty (BA), for plaque modification before the implantation of drug-eluting stents for patients with severely calcified coronary lesions. The ethics committees of People’s Hospital of Sichuan Province and West China Hospital of Sichuan University, China, approved the study protocol. All patients provided signed informed consent before the PCI, and the patients were free to withdraw from the study for any reason at any stage.

### Inclusion and exclusion criteria

From January 2010 to September 2014, at the Cardiovascular Departments of the People’s Hospital of Sichuan Province and West China Hospital of Sichuan University, China, we evaluated 80 consecutive patients with 80 severely calcified coronary lesions who were scheduled for RA, CB, or both prior to PCI and drug-eluting stent implantation. These patients met the following inclusion criteria of the study: with at least one severe coronary stenosis ≥70 %, as indicated by angiography; with at least one severely calcified lesion of the target vessels, defined as a calcium arc of ≥180° [24]; and agreed to receive an RA or CB-based intensive modification of the plaque before PCI.

Patients were excluded from the current study for any of the following: acute thrombosis with unstable hemodynamic status requiring emergent PCI; previous PCI and presence of in-stent restenosis; extremely tortuous or angulated lesions or lesions with dissection before balloon expansion; lesion within a vein graft; or other complications or comorbidities which were contraindicated for angiography or PCI (e.g., severe renal dysfunction).

### Randomization and treatment strategies

The included patients were randomized to either of 2 groups based on plaque modification strategies: pretreatment of the severely calcified plaque with RA and subsequent CB (RA + CB group); or pretreatment with RA and conventional plain balloon (RA group). The randomization was performed according to a random sequence generated by a computer program. No blinding was adopted in this study. The PCI procedures and the IVUS examinations were performed by a group of experienced physicians of interventional cardiology.

### PCI procedures

After confirmation of the diagnosis of coronary heart disease, all of the included patients were pretreated with aspirin or clopidogrel. A 300-mg loading dose of clopidogrel was administered before the procedure if patients were not pretreated. If they were considered to have a heavy thrombotic burden, periprocedural glycoprotein IIb/IIIa inhibitors were used, at the operator’s discretion.

During interventions, intravenous unfractionated heparin (100 IU/kg) was administered to maintain activated clotting time ≥300 s. A digital cardiovascular Artis zee III ceiling-mounted Siemens system was applied for the angiographic and PCI process. The choice of interventional strategy and size of the sirolimus drug-eluting stent were left to the discretion of the same operator for all patients.

An oral antiplatelet therapy regimen was implemented in accordance with the current guidelines, i.e., a combination of aspirin and clopidogrel at least 12 months for drug-eluting stent [16, 25]. After the PCI, cardiovascular medications were prescribed depending on the comorbidities of the patients, and the post-procedural medical therapy often included aspirin, clopidogrel, statin, beta-blocker, angiotensin-converting enzyme-inhibitors, and angiotensin-receptor blockers.

### Description of RA and CB processes

For patients in the RA + CB group, we pretreated severely calcified plaque with RA, and then subsequently with RA, with pre-dilation of the lesion achieved with a CB. As for patients randomized to the RA group, the calcified lesion was pretreated with RA but only a conventional plain balloon was selected for subsequent pre-dilation.

RA was performed with a burr size of 1.25, 1.50, or 1.75 mm, and was selected to reach a burr/vessel ratio of 0.5 (maximum: 0.7 if needed). The rotational speed of the burr ranged from 140,000 to 180,000 rotations per minute. The burr was platformed immediately proximal to the lesion to avoid injury to the healthy vessel segment. Intracoronary heparin and nitroglycerin were used to prevent any slow-flow occurring during or after RA.

For patients in both groups, the diameter of the CB or the plain conventional balloon selected for pre-dilatation was ≤0.5 mm that of the planned stent size, based on the vessel media-to-media diameters determined by IVUS. Stents were deployed after confirmation of full balloon expansion. Non-compliant balloon postdilatation after stenting was considered when IVUS revealed poor stent expansion.

### IVUS procedures

After conventional coronary angiography, IVUS was performed at the baseline timepoint and repeated immediately after stent implantations in all cases. The IVUS examinations were performed using a Boston Scientific image processor iLab Ultrasound Imaging System. Briefly, the IVUS catheter was carefully advanced distal to the culprit lesion under fluoroscopic guidance, and was then withdrawn automatically at 0.5 mm/s to perform the imaging sequence, which started 20 mm distal to the lesion and ended at the aorto-ostial junction. For those patients with severely narrow lesions in which IVUS could not access the distal segment even after pre-dilatation, the minimum lumen cross-sectional area (CSA) was estimated as the lumen CSA of the arrival section.

### IVUS analysis

Off-line IVUS analyses were performed with the use of Planimetry software (echoPlaque 3.0, Indec Systems). Quantitative IVUS measurements included the diameters and the CSA of the stent and the external elastic membrane. The IVUS measurements were evaluated based on the American College of Cardiology Clinical Expert Consensus Document on Standards for Acquisition, Measurement, and Reporting of Intravascular Ultrasound Studies [26].

After stent implantation, we measured the minimum stent CSA, and the acute lumen gain was estimated as the minimum intra-stent CSA after stenting minus the minimum CSA before stenting.

### Outcomes and clinical follow-up of the patients

The primary outcome of the study was the acute lumen gain of the patients in the 2 groups. Also analyzed were the RA-associated periprocedural rates of successful stent implantation and periprocedural complications (including coronary dissection, coronary perforation, coronary slow flow or no-reflow, stent thrombosis, and stent dislodgment), balloon dilation, and PCI.

The patients were followed for at least one year after the PCI, and another angiographic examination was performed. Major adverse cardiovascular events (MACE) were documented including death, myocardial infarction, and target vessel revascularization. Death included all-cause mortality. Myocardial infarction was defined according to current guidelines [27]. Target vessel revascularization was defined as revascularization during the follow-up period due to restenosis, either within the target lesion or within the same coronary artery.

### Statistical analyses

Continuous data are presented as the mean ± standard deviation and the categorical data are presented as number and percentage. Each set of data was subjected to a test for normal distribution. Differences in the continuous and categorical data between the 2 groups were analyzed using Student’s *t*-test or chi-squared analysis. Statistical analyses were achieved with SPSS 16.0 software (SPSS, Chicago, IL, USA). A *P*-value < 0.05 was considered statistically significant.

## Results

### Demographic and clinical baseline characteristics of the patients

Initially, 80 patients with coronary heart disease with severely calcified lesions in the target arteries and scheduled to receive a selective PCI were enrolled in the study, and were apportioned equally to the RA + CB group or the RA group. However, 5 patients in the RA + CB group and 4 patients in the RA group later withdrew from the study, for personal reasons. Finally, 71 patients (35 in the RA + CB group and 36 in the RA group) were included in the current analyses.

The mean age of the patients was 71.5 years, and there were 50 men and 21 women (Table 1). All of the included patients successfully underwent the IVUS examination and PCI, and were followed for at least 1 year. The RA + CB and RA groups were similar for baseline characteristics including age, gender, coronary heart disease diagnosis and risk factors, comorbidities and past history of PCI and coronary artery bypass graft (*P* > 0.05, all), except that the prevalence of chronic kidney disease was significantly higher in the RA + CB group (28.6 %) than in the RA group (8.3 %; *P* = 0.027).

**Table 1:** Baseline demographic clinical characteristics of patients in the RA + CB and RA (control) groups

		RA + CB	RA	*P*
Subjects, *n*		35	36	
Demographics	Age, y	69.3 ± 11.6	72.2 ± 10.2	0.269
	Male, n (%)	25 (71.4)	25 (69.4)	0.855
	Body mass index, kg/m^2^	26.2 ± 4.9	24.8 ± 4.1	0.184
Diagnosis, n (%)	Stable angina	7 (20.0)	6 (16.7)	0.717
	Unstable angina	26 (74.3)	28 (77.8)	0.730
	Acute myocardial infarction	2 (5.7)	2 (5.6)	0.977
Risk factors, n (%)	Diabetes mellitus	25 (71.4)	27 (75.0)	0.734
	Hypertension	27 (77.1)	28 (77.8)	0.949
	Current smokers	20 (57.1)	22 (61.1)	0.734
	Dyslipidemia	18 (51.4)	20 (55.6)	0.727
	Chronic kidney disease	10 (28.6) *	3 (8.3)	0.027
	LVEF < 50 %	14 (40.0)	16 (44.4)	0.705
Past history, n (%)	Myocardial infarction	8 (22.9)	10 (27.8)	0.738
	PCI	5 (14.3)	6 (16.7)	0.782
	Coronary artery bypass graft	0 (0)	0 (0)	–

*P < 0.05 compared with RA group

### Coronary lesions and the PCI procedures

The RA + CB and the RA groups were well matched for the numbers of affected arteries, the prevalence of bifurcated, ostial, and chronic total occlusions, and the Medina classifications of the bifurcated lesions (Table 2). However, the percentage of patients with a left main arterial lesion was significantly higher in the RA + CB group (22.9 %) than the RA group (5.6 %, *P* = 0.036). The two groups were also similar with regard to reference vessel diameter, minimal lumen diameter, total lesion length, and calcium length ratio, as evaluated by IVUS before PCI (Table 3).

**Table 2:** Characteristics of coronary lesions and PCI procedures of patients in the RA + CB and RA (control) groups*

		RA + CB	RA	*P*
Subjects, n		35	36	
Lesion location, n (%)	Left main coronary artery	8 (22.9) *	2 (5.6)	0.036
	Left anterior descending artery	20 (57.1)	23 (63.9)	0.561
	Left circumflex artery	5 (14.3)	4 (11.1)	0.688
	Right coronary	7 (20.0)	9 (25.0)	0.614
	Ostial lesion	3 (8.6)	4 (11.1)	0.720
	Chronic total occlusion	0 (0)	0 (0)	–
	Bifurcated lesion (Medina 1, 1, 1)	11 (31.4)	14 (38.9)	0.511
	Bifurcated lesion (Medina 1, 1, 0)	2 (5.7)	2 (5.6)	0.977
Indications for RA	Balloon catheter not passed, n (%)	20 (57.1)	22 (61.1)	0.275
	Balloon catheter under expansion, n (%)	15 (42.9)	14 (38.9)	0.327
	Final bur size, mm	1.58 ± 0.15	1.51 ± 0.18	0.100
	Burr/artery ratio	0.56 ± 0.03	0.56 ± 0.04	0.934
Pre-dilatation after RA	Pre-dilatation, n (%)	35 (100)	36 (100)	1.000
	Max balloon diameter, mm	2.65 ± 0.31	2.53 ± 0.37	0.163
	Max balloon inflation pressure, atm	13.8 ± 1.3	14.1 ± 1.0	0.190
	Balloon inflation time, s	18.3 ± 3.6	19.7 ± 4.0	0.134
Stent implantation	Stent delivery failure, n (%)	0 (0)	0 (0)	__
	Stent diameter, mm	2.79 ± 0.29	2.73 ± 0.37	0.418
	Stent release pressure, atm	14.5 ± 1.5	14.8 ± 1.5	0.377
Post-dilatation after stent	Non-compliant balloon, n (%)	28 (82.9)	31 (86.1)	0.188
	Max balloon inflation pressure, atm	18.4 ± 1.7	18.8 ± 2.4	0.421
	Balloon inflation time, s	20.4 ± 2.9	19.5 ± 2.4	0.210

*P < 0.05 compared with RA group

**Table 3:** Quantitative coronary angiography (QCA) and intravascular ultrasonographic (IVUS) analyses of lesion characteristics

		RA + CB	RA	*P*
	Subjects, n	35	36	
Baseline	Total lesion length, mm	31.3 ± 13.6	29.5 ± 13.1	0.566
	Calcium length ratio	0.81 ± 0.10	0.76 ± 0.12	0.093
	Superficial calcium arc	288.1° ± 36.3°	279.7° ± 41.8°	0.363
	Reference vessel diameter, mm	2.8 ± 0.3	2.7 ± 0.4	0.253
	Min. lumen diameter, mm	1.3 ± 0.2	1.3 ± 0.2	0.869
	Reference vessel CSA, mm^2^	6.4 ± 1.5	6.0 ± 1.9	0.315
	Min. CSA, mm^2^	1.3 ± 0.4	1.3 ± 0.4	0.896
	Lumen stenosis rate, %	79.1 ± 4.8	77.8 ± 4.2	0.217
After stent	Total stent length per lesion, mm	38.5 ± 13.1	35.8 ± 12.2	0.365
	Min. stent diameter, mm	2.7 ± 0.4	2.5 ± 0.4	0.097
	Min. stent CSA, mm^2^	5.9 ± 1.7 *	5.0 ± 1.4	0.021
	Residual lumen stenosis rate, %	10.5 ± 10.4	14.7 ± 10.8	0.103
	Acute CSA gain, mm^2^	4.5 ± 1.5 *	3.8 ± 1.5	0.035

*Min* minimum, *CSA* cross sectional area

*P < 0.05 compared with RA group

All of the included patients received modification of the calcified lesions with RA, and the two groups were not significantly different with regard to the final burr size, or the burr-to-artery ratio (Table 2). After RA, pre-dilations of the lesions were performed in patients of the RA + CB and RA groups with CB and conventional plain balloons, respectively. Balloon underexpansion was adequately resolved since repeated RA was performed in patients with balloon underexpansion, and no residual balloon underexpansion existed for the included patients in either group. No significant difference was detected for maximal balloon diameters, maximal balloon inflation pressures, or mean balloon inflation time for patients randomized to the two groups (Table 2).

All of the patients in both groups received successful stent delivery. Similarly, the two groups were matched for mean stent diameters, lengths, and stent release pressures (Table 3). The two groups were comparable for the proportions of patients who received post-dilations with non-compliant balloons, the maximal balloon inflation pressures, and the mean balloon inflation time (Table 2).

### Gain in acute CSA after PCI

The IVUS-evaluated mean arc of superficial calcium and baseline minimum lumen CSA of the RA + CB group (288.1 ± 36.3° and 1.3 ± 0.4 mm^2^, respectively) were statistically similar to that of the RA group (279.7 ± 47.5° and 1.3 ± 0.4 mm^2^; *P* > 0.05, both; Table 3). However, the minimum stent CSA after PCI of the RA + CB group (5.9 ± 1.7 mm^2^) was significantly larger than that of the RA group (5.0 ± 1.4 mm^2^; *P* = 0.021). More importantly, patients in the RA + CB group achieved a significantly larger acute CSA gain after PCI (4.5 ± 1.5 mm^2^) than did patients in the RA group (3.8 ± 1.5 mm^2^; *P* = 0.035). This suggests that RA combined with CB may be more effective for severely calcified lesions than RA with the conventional balloon.

### Periprocedural complications and follow-up results

No patient in either group experienced periprocedural complications, including coronary perforation, stent thrombosis, or stent dislodgment (Table 4). Three patients in each group suffered from coronary slow flow or no-reflow; this rate was not significantly different between the 2 groups. Coronary dissection occurred in 3 patients in the RA + CB group (8.3 %) and 4 patients in the RA group (11.1 %, *P* > 0.05). These results suggest that modification of plaques with RA and CB did not carry a higher risk of periprocedural complications than RA with the conventional balloon.

**Table 4:** Incidences of periprocedural complications of patients in the RA + CB and the RA control groups, *n* (%)

	RA + CB	RA	*P*
Subjects, n	35	36	
Coronary dissection	3 (8.3)	4 (11.1)	0.720
Coronary perforation	0 (0)	0 (0)	–
Coronary slow flow or no-reflow	3 (8.6)	3 (8.3)	0.972
Stent thrombosis	0 (0)	0 (0)	–
Stent dislodgment	0 (0)	0 (0)	–

After a mean follow-up of 13.2 ± 4.7 months, no myocardial infarction, stroke, or cardiovascular death occurred in patients of either group (Table 5). However, the percentage of patients who received revascularization for restenosis of the target vessel was significantly higher in the RA group (22.2 %) than the RA + CB group (5.7 %, *P* = 0.046) by the one-year follow-up.

**Table 5:** Outcomes during the 1-year follow-up of patients in the RA + CB and RA control groups, *n* (%)

	RA + CB	RA	*P*
Subjects, n	35	36	
Non-fatal myocardial infarction	0 (0)	0 (0)	–
Non-fatal stroke	0 (0)	0 (0)	–
Cardiovascular death	0 (0)	0 (0)	–
Target lesion revascularization	2 (5.7) *	8 (22.2)	0.046
MACE	2 (5.7) *	4 (22.2)	0.046

*P < 0.05 compared with RA group

## Discussion

**Fig. 1:**
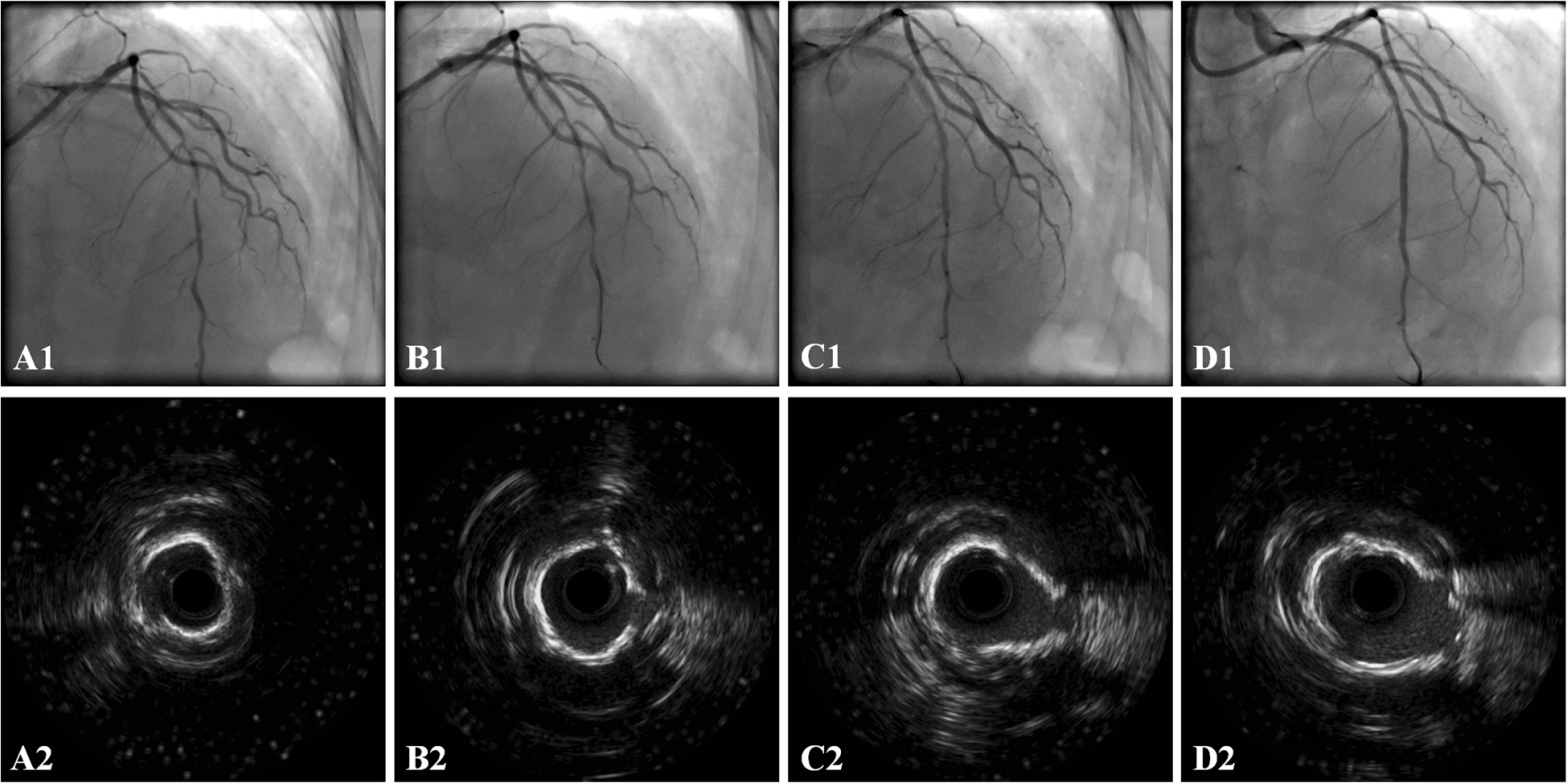
The angiography and intravascular ultrasound (IVUS) images of severe coronary calcification lesion before and after rotational atherectomy, cutting balloon, and stent deployment. A1 and A2, Severe calcific stenosis of the *left* anterior descending artery; B1 and B2, post-rotablator; C1 and C2, post-cutting balloon; D1 and D2, post-stent

This pilot randomized controlled trial evaluated the efficacy and safety of RA combined with CB for intensive plaque modification in patients with severely calcified coronary lesions, relative to RA with conventional balloon. We found that in these patients, RA combined with CB was associated with significantly greater acute CSA gain (Fig. 1), and RA and CB did not carry a higher risk of periprocedural complications during the subsequent PCI. This indicates that plaque modification with RA and CB was safe for these patients. Regarding clinical outcomes, at the 1-year follow-up the revascularization for stenosis of the target vessel, and MACE, associated with RA and CB was lower than that of RA with conventional balloon. This suggests that RA combined with CB may lead to a better clinical prognosis.

Improved acute CSA gain may be a protective factor that prevents restenosis and MACE after PCI [28]. Our results indicate that for patients with severely calcified coronary lesions, intensive plaque modification with RA and CB was safe, and was associated with improved immediate PCI results compared with the RA and conventional balloon. Although the potential therapeutic role of combined plaque modification with RA and CB for patients with severely calcified coronary lesions has been suggested in previous studies [11], to the best of our knowledge this is the first randomized controlled trial that confirms the efficacy and safety of this treatment strategy under these circumstances.

A case report published in 2004 [29] reported successful plaque modification via RA and CB prior to stent implantation in a 61-year-old man with severe calcified plaque in the ostium of the left circumflex of the left coronary artery. The combined RA and CB strategy facilitated optimal stent deployment. A recent retrospective cohort study [23] showed that RA prior to CB plaque modification for drug-eluting stent implantation in severely calcified lesions appeared to be more efficacious than RA with a plain balloon, with significantly larger final stent CSA. The results of our present study further confirmed the previous clinical observations that intensive plaque modification with RA and CB was associated with an improved acute CAS gain after PCI [23] —the combined plaque preparation strategy helped optimize the PCI, particularly the deployment of the stents in these patients with severely calcified coronary lesions.

Previous evidence showed that poor acute CSA gain after PCI may be a predictor of increased risk for long-term MACE [28]. The present study may be clinically important, since it shows that intensive plaque modification with RA and CB was associated with a lower risk of target lesion revascularization. However, whether combined plaque modification with RA and CB is associated with better long-term clinical outcomes compared with RA with a plain balloon in patients with severely calcified coronary lesions still needs to be determined in further studies.

Combined RA and CB plaque modification on acute luminal gain for these patients may be of improved benefit, compared with RA with a plain balloon, because it overcomes the disadvantages of applying either RA or CB alone [9, 22]. Pretreatment with RA can be limited by the maximum burr size (1.75 mm), which may be too small for some severely calcified lesions in vessels of relatively large diameter; concurrent application with CB-based plaque modification may further improve patency of these vessels. The CB catheter is relatively stiff and non-compliant, which limits its movements through lesions with sharp angulations. In the present study, use of the CB was associated with better-controlled pre-dilation of the calcified lesion, which reduced the risk of extensive dissection caused by stretch of the arterial wall when using the conventional plain balloon.

Interestingly, pretreatment with RA may facilitate negotiation with the CB catheters, thereby enhancing the efficacy of the CB technique. Plaque modification with RA and CB can optimize the deployment of the stents, although this is also dependent on the skill of the operator, to improve the acute PCI outcome and reduce the chances of early complications such as stent restenosis or thrombosis induced by stent underexpansion. Obviously, the exact mechanisms underlying the immediate benefits of combined lesion preparation with RA and CB deserve further investigation.

Although it has been suggested that RA and CB carries higher risks of periprocedural complications such as coronary dissection or perforation, in the present study we found that RA combined with CB was not associated with increased risk compared with RA with a plain balloon. Moreover, the group that received RA and CB had a lower rate of revascularization for restenosis of the target vessel and MACE during the 1-year follow-up, indicating that RA and CB may lead to better clinical prognosis.

Our study is limited in that it is a pilot clinical trial, the sample size was small, and the follow-up duration was short. Further large-scale randomized controlled trials are needed to confirm our results and explore the potential benefits of aggressive plaque modification with RA combined with CB for calcified lesions. In addition, this study was designed as an open-label trial and the baseline characteristics were not completely similar between the 2 groups. Yet, since significantly more patients in the RA + CB group had chronic kidney disease, and significantly more had lesions of the left main coronary artery, and both are known predictors of poor PCI outcomes, these imbalances did not confound the results. Finally, due to the small sample size we were unable to perform a subgroup analysis to explore whether patients with certain characteristics could benefit more from the combined plaque modification strategy. Future studies may be needed to clarify these questions.

## Conclusion

This pilot randomized controlled trial indicated that aggressive plaque preparation with combined RA and CB seems to be safe and effective for patients with severely calcified coronary lesions, and is associated with better acute PCI results and clinical outcomes compared with RA with a conventional balloon. Future studies are needed to confirm our results and explore the potential long-term benefits of aggressive plaque modification with RA combined with CB for calcified lesions.

## Abbreviations

CB: cutting balloon
CSAs: cross-sectional areas
IVUS: intravascular ultrasound
MACE: major adverse cardiovascular events
Min: minimum
PCI: percutaneous coronary intervention
RA: rotational atherectomy
